# Video-assisted minimal access surgery for complicated mitral valve endocarditis, tricuspid valve insufficiency and progressive coronary disease after previous CABG - in the time of COVID-19: a case report

**DOI:** 10.1186/s13019-021-01517-8

**Published:** 2021-06-24

**Authors:** Terézia B. Andrási, Nunijiati Abudureheman, Alannah Glück, Maximilian Vondran, Gerhard Dinges, Ildar Talipov, Ardawan J. Rastan

**Affiliations:** 1 Department of Cardiac Surgery, Philipps University of Marburg, Baldingerstrasse 1, 35039 Marburg, Germany; 2 School of Medicine, Philipps University of Marburg, Marburg, Germany; 3 Department of Anesthesiology and Intensive Care Medicine, Philipps University of Marburg, Marburg, Germany

**Keywords:** Minimally invasive video-assisted cardiac surgery, Valve endocarditis, Coronary revascularization, Cerebral septic embolism, Covid-19

## Abstract

**Background:**

The timing for heart surgery following cerebral embolization after cardiac valve vegetation is vital to postoperative recovery being uneventful, additionally Covid-19 may negatively affect the outcome. Minimally invasive methods and upgraded surgical instruments maximize the benefits of surgery also in complex cardiac revision cases with substantial perioperative risk.

**Case presentation:**

A 68 y.o. patient, 10 years after previous sternotomy for OPCAB was referred to cardiac surgery on the 10th postoperative day after neurosurgical intervention for intracerebral bleeding with suspected mitral valve endocarditis. Mitral valve vegetation, tricuspid valve insufficiency and coronary stenosis were diagnosed and treated by minimally invasive revision cardiac surgery on the 14th postoperative day after neurosurgery.

**Conclusion:**

The present clinical case demonstrates for the first time that the minimally invasive approach via right anterior mini-thoracotomy can be safely used for concomitant complex mitral valve reconstruction, tricuspid valve repair and aorto-coronary bypass surgery, even as a revision procedure in the presence of florid endocarditis after recent neurosurgical intervention. The Covid-19 pandemic and prophylactic patient isolation slow down the efficacy of pulmonary weaning and mobilisation and prolong the need for ICU treatment, without adversely affecting long-term outcome.

## Background

The beginnings of minimally invasive thoracic surgery are attributed to Hans Christian Jacobaeus [1] and the first developments of suitable equipment were thoroughly described by Larry R. Kaiser in 1994 [2].

After decades of slow development of minimal-invasive surgical technologies in the twentieth century, significant innovations in the field of cardiac surgical procedures were marked in the 1990s by Carpentier’s first video-assisted minimally invasive mitral valve surgery via mini-thoracotomy [3] and Chitwood’s transthoracic aortic clamp [4]. Few years later, Mohr et al. [5] developed a video-assisting port access technology that allowed better visualization of the atrioventricular valves and reduced both CPB and cross-clamp times.

Although double atrioventricular valve procedures are successfully performed via right mini thoracotomy [6], the suitability of minimally invasive approaches treating endocarditis, revision procedures and combined coronary and valve procedures, remains debatable.

Herein, we describe a complex case of successful double valve repair and coronary bypass implantation using minimal-invasive approach in a patient aged 68 with mitral valve endocarditis after having received a sternotomy treating CABG 20 years ago, and complicated by recent neurosurgical intervention for cerebral septic embolism. The postoperative course was impeded by fungal pneumonia and suspicion of Covid-19 infection.

## Case presentation

**Fig. 1:**
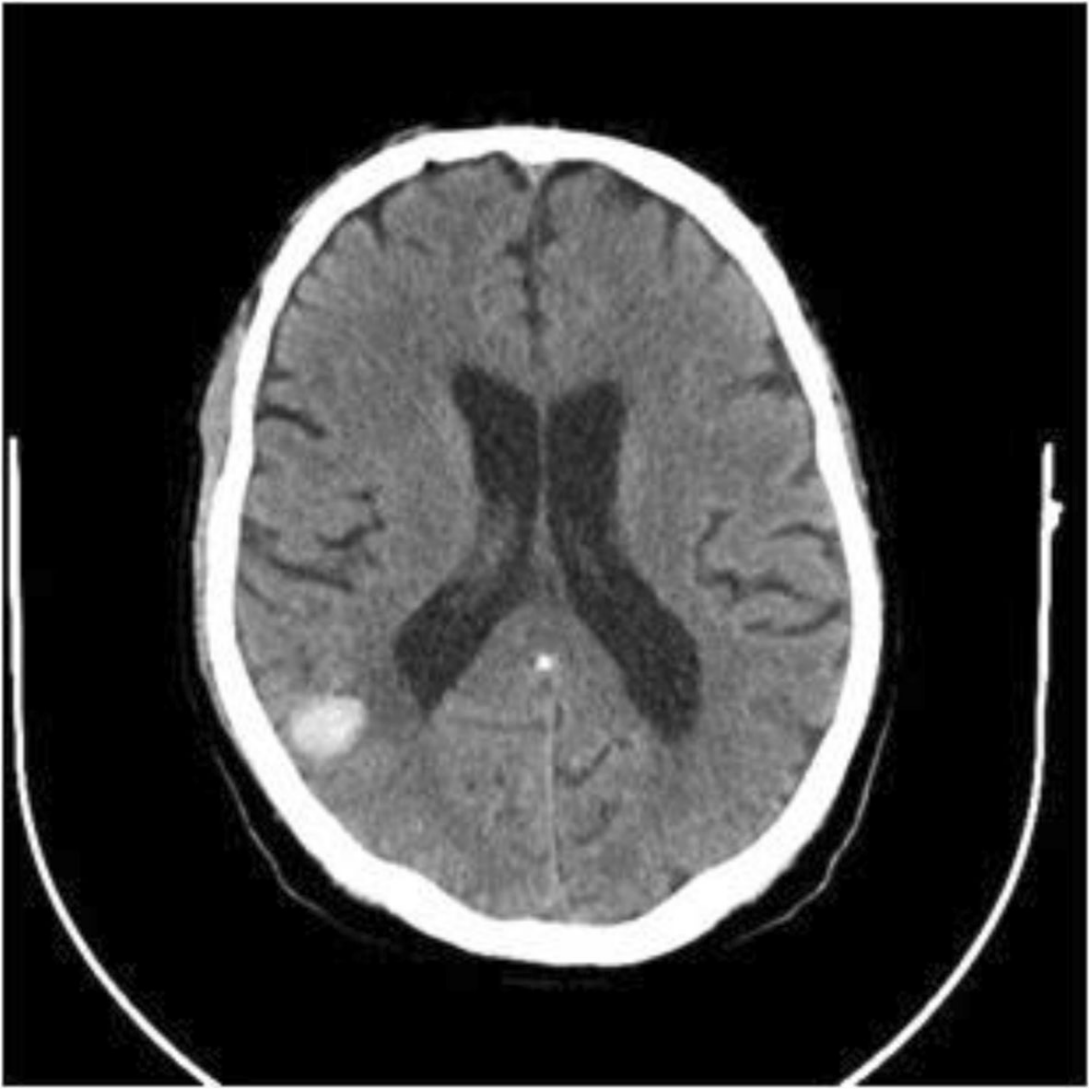
Preoperative Cranio-Computer-Tomography Scan. Right parietal 8 × 15 × 12 mm mass

**Fig. 2:**
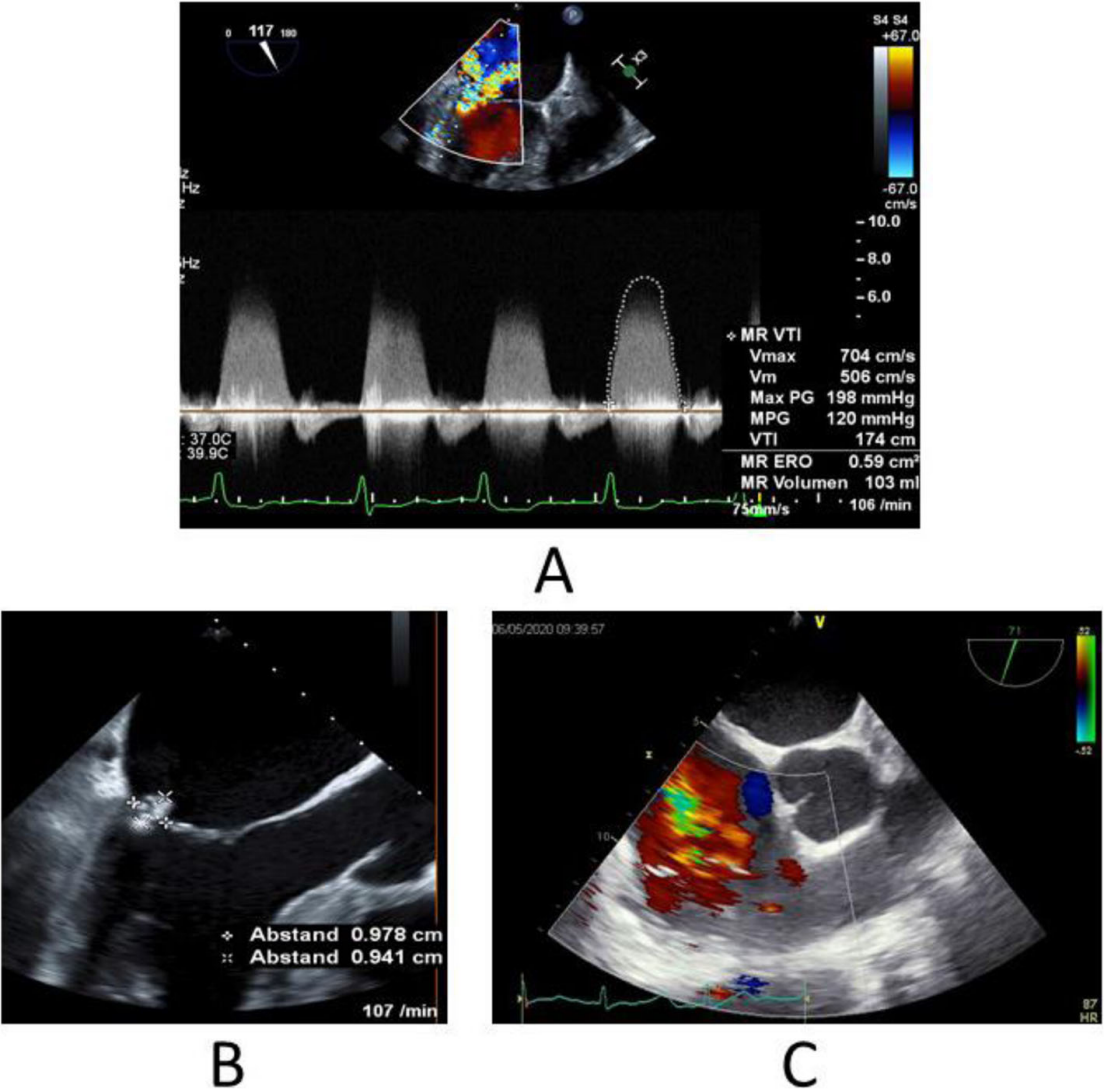
Preoperative Echocardiography. **a** Mitral valve regurgitation; b 10 × 12 mm vegetation of the mitral valve; **c** tricuspid valve regurgitation

**Fig. 3:**
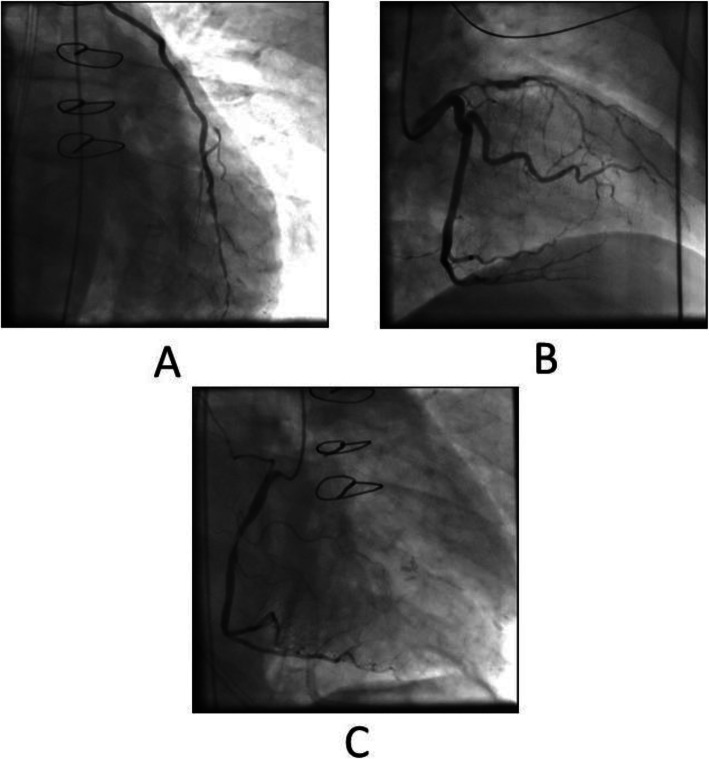
Preoperative Coronary Angiography. **a** patent LIMA-LAD Bypass; **b** high-degree proximal LAD Stenosis and normal RCX Vessel; **c** 90% Stenosis of the RCA

A 68-year-old diabetic male patient was urgently admitted to our hospital due to progressive changes in consciousness, ten years after having had OPCAB surgery (LIMA-LAD Bypass) via full sternotomy. The cranio-CT revealed a hemorrhagic metastasis-suspicious mass (8 × 15 × 12 mm) in the right superior temporal gyrus on the posterior wall of the Sylvian fissure (Fig. 1). Neurosurgery was performed by microsurgical technique. On the 10th postoperative day, the patient developed sepsis with cardiopulmonary decompensation and cardiorenal syndrome requiring dialysis. The transesophageal echocardiography revealed high-degree mitral valve regurgitation with an eccentric jet flow (Fig. 2a), a 10 × 12 × 12 mm vegetation adherent to the P2 and P3 posterior leaflet segments (Fig. 2b), and tricuspid valve regurgitation grade II with systolic reflux in the pulmonary veins (Fig. 2c). The coronary angiography revealed a patent LIMA-LAD Bypass (Fig. 3a), normal RCX (Fig. 3) and a relevant newly developed 70% stenosis of the RCA in segment 2 (Fig. 3c). After signed consent was obtained, the patient was transferred to the cardiothoracic surgery department and underwent complex minimally invasive cardiac repair on the 14th postoperative day after neurosurgery.

### Surgical procedure

Standard general anesthesia was applied. A saphenous vein segment was endoscopically harvested from the left thigh. CPB was initiated after cut-down cannulation of the right femoral artery and vein. The heart was approached via a 6 cm skin incision over the fifth right intercostal space from the anterior to medial axillary line. The pericardium was longitudinally incised 3 cm above the phrenic nerve. A needle vent was placed in the ascending aorta. The camera optics for video-assisted surgery and the Chitwood clamp were prepared through separate stab incisions. Aortic cross-clamping and cardioplegic arrest were instituted. The LIMA-LAD Bypass was not visualized and remained opened during the entire procedure. Hyperkalemic cardiac arrest was induced with cold crystalloid cardioplegic solution and was maintained without difficulties during the procedure performed under mild hypothermia (32 °C).

#### Step 1: complex mitral valve reconstruction on CPB with cardiac arrest

**Fig. 4:**
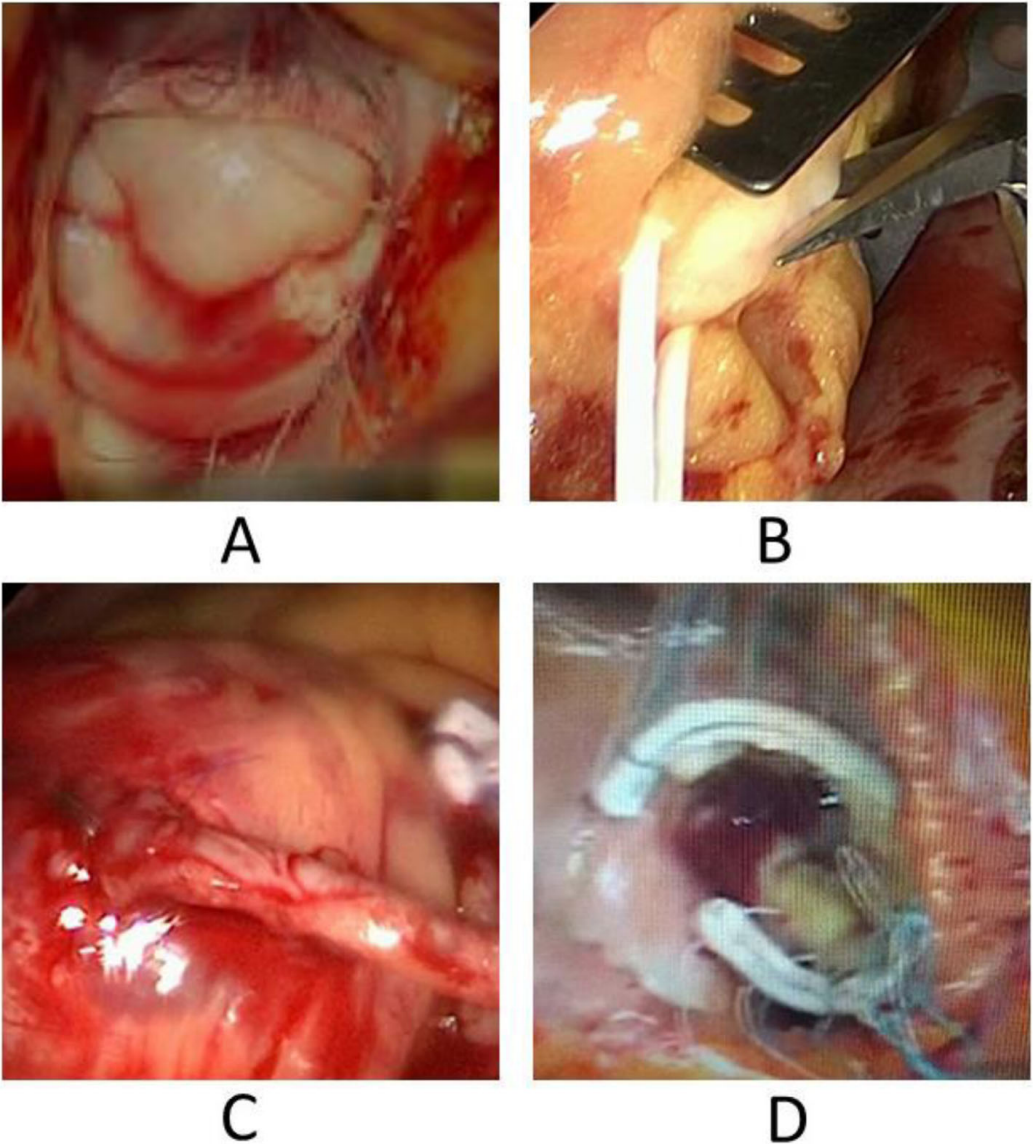
Operative procedure. **a** mitral valve reconstruction; **b** distal bypass anastomosis on the RCA; **c** proximal aortic bypass anastomosis; **d** Tricuspid valve annuloplasty

The mitral valve was exposed and inspected through a left atrial incision. The vegetation was removed. An annulus dilatation, endocarditic destruction and flail of the P3 segment were assessed and treated by quadrangular P2-P3 resection, sliding plasty and ring-annuloplasty (Carpentier Edwards Physio Ring 30 mm, Fig. 4a). The left atrium was closed after the successful water test of the valve function.

#### Step 2: Aorto-coronary venous bypass to the RCA on CPB with cardiac arrest

The RCA was exposed and incised on the crux (Fig. 4b). The aorto-coronary bypass (Fig. 4c) was implanted during cardiac arrest. Aortic declamping followed and reperfusion was initiated after 185 min of cardiac arrest. Epicardial temporary pacemaker stimulation was applied.

#### Step 3: tricuspid valve reconstruction on the beating heart

Vena cava inferior and superior were clamped and total bypass instituted. The right atriotomy was made on the beating heart on CPB during rewarming in the mid right atrium. A 30 mm Cosgrove-Edwards Band was implanted (Fig. 4d) after accurate sizing. The atriotomy was closed in two layers and total bypass resumed after 32 min.

### Resuming CPB and end of surgery

**Fig. 5:**
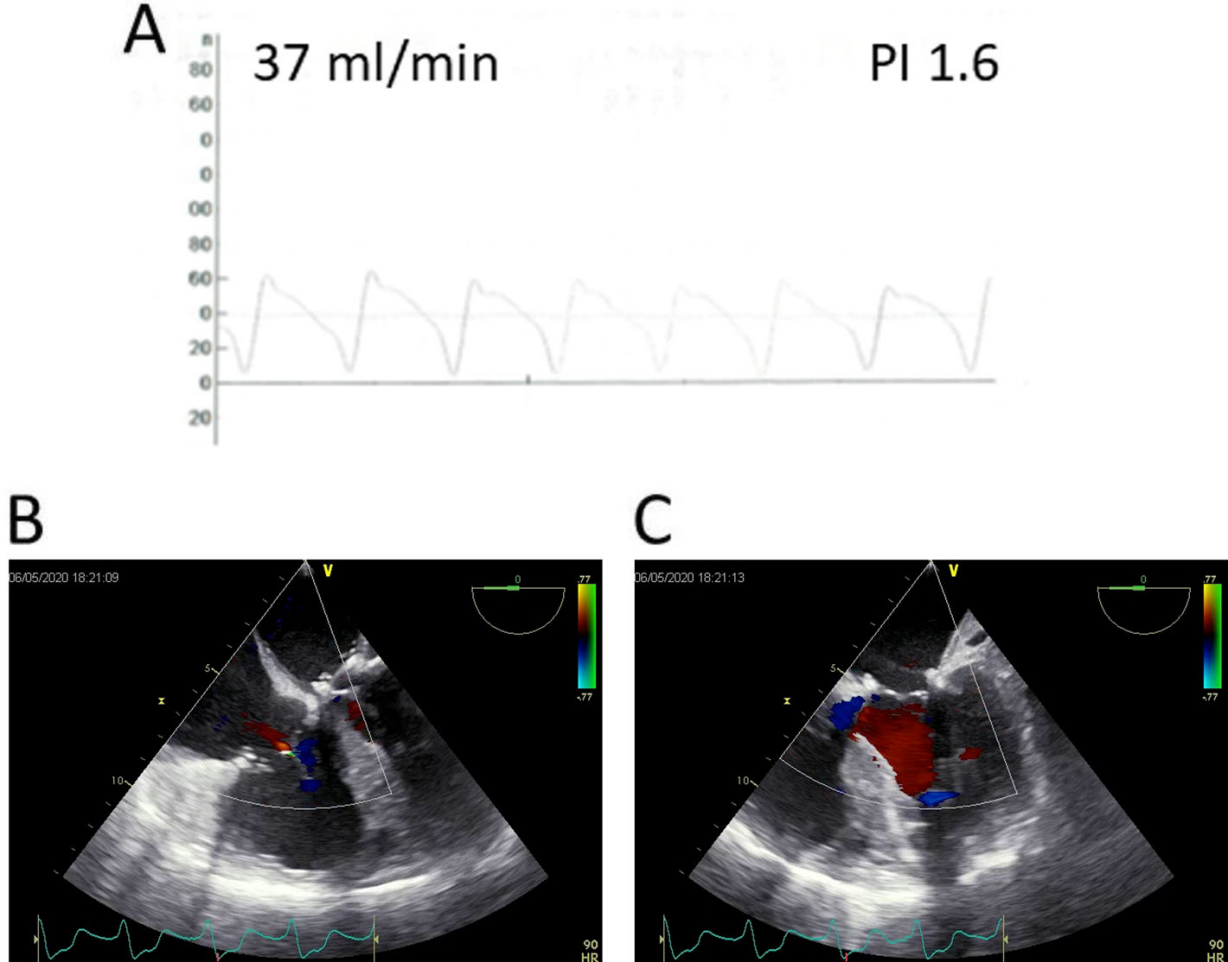
Postoperative quality assessment. **a** Bypass flow Measurement; **b** echocardiography of mitral valve function; **c** echography of tricuspid valve function

Transition from mechanical pump-assisted circulation to spontaneous heart activity was easily achieved with sufficient blood flow to maintain systemic circulation, under minimal catecholamine support. The transit time flow measurement of the RCA venous bypass revealed 37 ml/min (Fig. 5a). Transesophageal echocardiography showed normal systolic heart function and atrioventricular valve function without wall motion disturbances (Fig. 5b, c). Heparin was antagonized. Central aortic venting and CPB cannulas were removed from the groin. The pericardium was closed, and two chest drainage tubes positioned in the right pleura. The thoracotomy and groin incisions were closed in layers. The total operation time was 331 min.

### Postoperative recovery

**Fig. 6:**
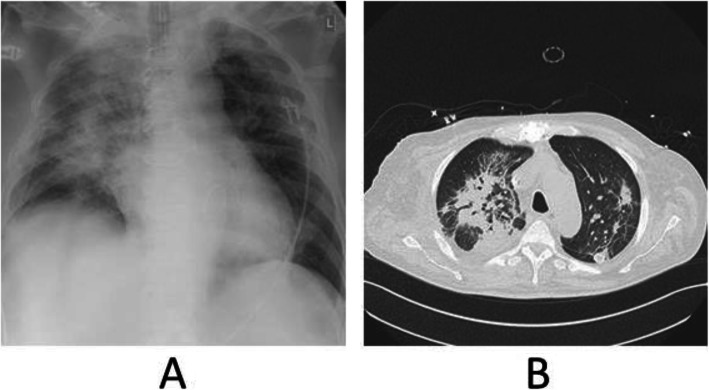
Suspicion of COVID-19 Infection. **a** Thoracic Radiography; **b** Thoracic CT-scan

Weaning was instituted and rush spontaneous breathing (CPAP ventilation) could be initiated in the early postoperative phase. After the operation, the patient came in contact with a later COVID-19-positive tested ICU nurse and a routine thoracic RTG examination (Fig. 6a) on the 2nd postoperative day (POD) raised the suspicion of Covid-19 infection. A thoracic CT-scan (Fig. 6b) was performed, and the patient was isolated. Routine testing was performed during the next 7 days. Fungal pneumonia was diagnosed, and a tracheotomy performed on the 9th POD, mobilization and feeding were initiated on the 16th POD after cardiac surgery. The patient was transferred to the rehabilitation center on the 27th POD breathing spontaneously without ventilator support. Full neurologic recovery was achieved on the 29th POD.

## Discussion and conclusion

Cerebral embolisation with underlying left heart valve endocarditis remains an indication for urgent cardiac repair. Nonetheless, open heart surgery early after neurosurgery in a patient with sepsis and multiorgan dysfunction (cerebral, cardiac, pulmonary, renal) is challenging. In addition, revision surgery after sternotomy increases the risk for surgical complications and anticoagulation. Also, prolonged major surgery (double valve repair and aortocoronary bypass) under sepsis may further increase the risk for postoperative cerebral and thoracic bleeding, systemic inflammatory response and multiorgan failure.

Nonetheless, minimally invasive approaches have been shown to reduce the risk of major cardiac surgery, [6] although they are not yet validated for complex cardiac revision surgery and complicated endocarditis.

The present case demonstrates the suitability of a right lateral mini thoracotomy for the first time. It allows adequate exposure and handling of both atrioventricular valves and of the right coronary artery in a revision setting.

As previously described [7], the patent and opened LAD bypass did not cause any difficulties for rush cardiac arrest and reperfusion. The exposure of both atrioventricular valves can be easily achieved via right lateral mini-thoracotomy, and video-assistance allows adequate anatomic reconstruction of the valves (Fig. 4) with outstanding postoperative results (Fig. 6). The implantation of a bypass on the right coronary can be performed without any difficulties in combination with a valve implantation as also described above [8].

Therefore, the present case reveals not only the advantages of minimal access surgery such as limited adhesion preparation, reduced risk of hemodynamic instability, better wound healing and feasibility for tracheostomy, but also a safer outcome due to reduced surgical trauma, less bleeding and need for transfusion, lower risk for wound infections, less pain and favorable cosmesis. Additionally, faster recovery of pulmonary function, early mobilization, shorter hospital stays and decreased healthcare costs, make this a preferred approach for the surgeons.

The present clinical case demonstrates that the advantages arising from the procedural features enable the minimally invasive performance to generate a good outcome in complicated high-risk cardiac revision surgery.

The COVID-19 pandemic and prophylactic patient isolation significantly slowed down the efficacy of pulmonary weaning and mobilisation, and prolonged the demand for ICU treatment, did not however heighten postoperative morbidity by increasing dysfunction of other organs and systems.

In conclusion, the present case demonstrates that the minimally invasive approach can safely be used in complicated cases of mitral valve endocarditis with associated tricuspid and right coronary disease. Multimorbidity and previous coronary revascularisation are not contraindications, and a minimally invasive approach might minimize the risk for intracranial bleeding after recent neurosurgery. Aspects of minimal access surgery such as limited adhesion preparation, better wound healing, preservation of pulmonary function and feasibility for tracheostomy are advantageous. Efforts should be made to further develop these procedures, making their application available and safe for a larger population of patients.

## Data Availability

Data sharing is not applicable to this article as no datasets were generated or analysed during the current study.

## Abbreviations

CABG: Coronary artery bypass grafting
CPB: Cardiopulmonary bypass
CT: Computer tomography
LAD: Left anterior descending coronary artery
LIMA: Left internal mammary artery
OPCAB: Off-pump coronary artery bypass
POD: Postoperative day
RCA: Right coronary artery
